# A Simple Minimized System Based on Moving Drops for Antioxidant Analysis Using a Smartphone

**DOI:** 10.3390/molecules26195744

**Published:** 2021-09-22

**Authors:** Sutasinee Apichai, Kajorngai Thajee, Thanawat Pattananandecha, Chalermpong Saenjum, Kate Grudpan

**Affiliations:** 1 Department of Pharmaceutical Sciences, Faculty of Pharmacy, Chiang Mai University, Chiang Mai 50200, Thailand; sutasinee.apichai@gmail.com (S.A.); thanawat.pdecha@gmail.com (T.P.); 2 Cluster of Excellence on Biodiversity-Based Economics and Society (B.BES-CMU), Chiang Mai University, Chiang Mai 50200, Thailand; 3 Center of Excellence for Innovation in Analytical Science and Technology (I-ANALY-S-T), Chiang Mai University, Chiang Mai 50200, Thailand; nuibct@gmail.com; 4 Department of Chemistry, Faculty of Sciences, Chiang Mai University, Chiang Mai 50200, Thailand

**Keywords:** micro moving drop, flow analysis, downscaling chemical analysis, smartphone, antioxidant

## Abstract

In this paper, a novel antioxidant analysis is proposed using a simple minimized device based on moving drops as solution handling and a smartphone as a detector. This approach is based on the colorimetric determination of the scavenging activity against 2,2-diphenyl-1-picrylhydrazyl radical (DPPH^•^), expressed as the half-maximal inhibitory concentration (IC_50_), vitamin C equivalent antioxidant capacity (VCEAC), and Trolox equivalent antioxidant capacity (TEAC). A small drop of the positive control or the samples moves by eluting an ethanol drop down by the force of gravity to react with a DPPH^•^ drop in the detection zone. The color change of DPPH^•^ is monitored by a smartphone camera, and the color signals are processed using Adobe Photoshop software. The magenta-to-yellow ratio was successfully applied to evaluate the percentage of DPPH^•^ inhibition with no significant difference compared with the reference spectrophotometric method at a confidence level of 95%. The total phenolic content (TPC) was measured using the Folin–Ciocalteu assay. An application to Miang (fermented tea leaf extract) showed the consonant relationship between the scavenging activity of DPPH^•^ and TPC.

## 1. Introduction

The oxidation of free radicals in humans engenders cell damage, affecting the initiation and augmentation of various diseases such as cancer, gout, and atherosclerosis; cardiovascular and respiratory diseases; and aging. Antioxidant compounds have been shown to have the ability to inhibit the emergence and scavenge of free radicals in a variety of ways [1]. For example, they block oxidative enzymes, upregulate antioxidant enzymes, and directly respond to free radicals to minimize oxidative damage [2,3,4]. Thus, measuring total antioxidant capacity (TAC) is an important indicator expressed with various methods, especially the scavenging activity of free radical 2,2-diphenyl-1-picrylhydrazyl (DPPH^•^) and total phenolic content (TPC) [5].

Sustainable development shows an awareness of the importance of not causing irreversible harm to the future. By organizing the Sustainable Development Agenda, the United Nations promotes sustainable development. World leaders from various countries accepted the 17 sustainable goals as part of this agenda. Green analytical chemistry is also intended to encourage long-term sustainable development. This notion encompasses many techniques, including eliminating and reducing the number of chemical substances, avoiding toxic reagents, appropriately managing waste, and lowering energy consumption, all concerned with environmental friendliness, human health, and worker safety [6]. Miniaturized analytical methods and instruments are designed to reduce sample and reagent volumes, resulting in less waste formation, downscaling chemical analysis. Greener techniques would also include measurements with simplicity, rapidity, and directness. The speed of measurements could be improved by operating with multi-analyte and multi-parameter approaches [7]. Moreover, the approach leading to green analytical chemistry includes remote sensing using portable and cost-effective devices without extra power.

Flow injection analysis (FIA) was introduced as one of the solution-handling means for effective manipulation and measurement and for downscaling chemical analysis. Sequential injection analysis (SIA) was developed further by employing a syringe pump and a multiport selection valve for higher degrees of automation in solution handling with multi-reagent/sample handling capabilities [8]. Sequential injection (SI) systems with lab-on-valve (LIV) and SI with lab-at-valve (LAV) and lab-on-chip (LOC) were proposed for solution handling in the miniaturized chemical analysis [9]. They offer manipulation for microliter volumes of samples and reagents for a chemical analysis process. In addition to the pump and valve, a multifunctional pipette was used for simple micro-liquid handling in an LOC [10] that enabled contamination-free handling of the liquid and high throughput suitable for multi-analysis.

Several downscaling platforms have continuously been developed for antioxidant analysis to improve green analytical chemistry. The flow-based technique is one type of platform that gradually reduces bulky systems to small portable systems. Firstly, FIA, SIA, and LOC were developed for antioxidant analysis using a sample or reagent in the microliter volumes; however, such systems waste the carrier [11,12,13]. Then, the small, portable, environmentally friendly, and easy-to-use systems, suitable for on-site use in remote areas, were proposed, such as lab-on-paper and lab-on-thread. An analysis of antioxidant activity based on the scavenging activity of DPPH^•^ assay using lab-on-paper was reported. The grayscale intensity as a function of standard content was plotted to create a dose–response curve. Still, reports regarding the percentage of DPPH^•^ inhibition are lacking [14,15,16], with existing studies only reporting the percentage of hypochlorite scavenging activity using the well plate platform [17]. The report employed a smartphone as a detector similar to the proposed method in this work, but with different color property systems and the evaluation for the percentage of radical scavenging activity. A simple platform with moving drops concept is introduced as alternative to the well plate.

In this study, we employed a smartphone as a detector similar to the proposed method, but the suitable color signals were different for calculating the percentage of radical scavenging activity. However, the minimized platforms were of different manipulations and used different analytical volumes. In addition to the scavenging activity, TPC was measured using lab-on-paper and lab-on-thread, but sample loss into the material and non-homogenous color signals on the platform were found [18,19]. Although there was an effort to overcome this problem by modifying the fabrication of the platform, the fabrication was complex [20,21].

We adopted the previously reported platform for antioxidant analysis. The platform provides unique simple solution handling without external power and with a simple, cost-effective apparatus. This platform underlies the flow behavior in the microvolumes of the sample drop and reagent drops without carrier flow [22]. After sample and reagent drops are introduced into the platform using a multifunctional pipette, the drops can move with the gravity force along the sloping channel and collide themselves, causing suitable homogenous mixing. The operation of this miniaturized system reduces sample and reagent consumption. The system is operated without a pump, and homogeneous mixing occurs without external force. The system can be operated on-site in any place, even remotely, including rural areas with limited available power sources. Furthermore, it can overcome the problem of sample loss due to adsorption by a platform material, which occurs in other platforms: lab-on-paper and lab-on-thread. In addition, its fabrication is simple and low cost, and it provides good accuracy and reproducibility. These are directions toward green analytical chemistry [23].

## 2. Results and Discussion

### 2.1. Scavenging Activity on 2,2-Diphenyl-1-Picrylhydrazyl (DPPH^•^) Radicals

#### 2.1.1. The Kinetics of the 2,2-Diphenyl-1-Picrylhydrazyl Radical’s Scavenging Activity

The antioxidant activity in terms of the half-maximal inhibitory concentration (IC_50_), vitamin C equivalent antioxidant capacity (VCEAC), and Trolox equivalent antioxidant capacity (TEAC) was measured on the basis of DPPH^•^ scavenging. Scavenging through the reduction reaction of 2,2-diphenyl-1-picrylhydrazyl (DPPH^•^) to 2,2-diphenyl-1-picrylhydrazine (DPPH_2_) is measured on the basis of a colorimetric approach with color change from purple to yellow. Firstly, the kinetics of DPPH^•^ scavenging by the tested samples and positive control, namely, L-ascorbic acid, Trolox, and gallic acid, were investigated to find the steady state and optimum incubation period. The monitoring DPPH^•^ scavenging activity with the accurate initial time was possible by employing the proposed system as the capability of using a smartphone for recording the instant moment of the first touching of the drop of analyte and the drop of reagent. With the acceleration of the drops moving along the channels via gravity, when touching each other, mixing action was formed, without any other external force. The activity was calculated using the intensity of magenta and yellow colors from captured photography. In this study, the percentage of DPPH^•^ inhibition was calculated as follows (Equation (1)):

(1) $$\%I=\frac{I_{0}-I_{t}}{I_{0}}\;\times \;100$$

where *I_0_* and *I_t_* are the intensity ratios of magenta to yellow in the CMYK mode of the negative control and the antioxidant compound at the steady state, respectively.

From our investigation of the kinetics of DPPH^•^ scavenging activity by L-ascorbic acid, Trolox, and gallic acid, we found the highest activities with 3 min of an optimum incubation time, as shown in Figures S1–S3 (in the Supplementary Materials). The DPPH^•^ scavenging activity by Miang extract was slow and gradually stabilized at the incubation time of about 5–10 min, as shown in Figures S4–S6 (in the Supplementary Materials). At stable conditions, the percentages of DPPH^•^ inhibition by the different concentrations of L-ascorbic acid, Trolox, and gallic acid obtained from the proposed method and the reference method showed excellent linear correlations of 0.992, 0.997, and 0.998, respectively. Such correlation in the samples was also excellent, in the range of 0.994–0.999. The system proposed in this work provided a sample throughput of 10 samples/platform for the assays of the percentage of DPPH^•^ inhibition, while the system in the previous work [17] worked for 80 samples/platform with a new developed reagent. The platform system was reported for the assay of hypochlorite and percentage inhibition with a new reagent system. The proposed system in this work was operated using the common reagent for the purpose. The reagent can be commonly available, and the results can be referenced the conventional method. Apart from the assay for the percentage of DPPH^•^ inhibition, the proposed platform can also be used for IC_50_, VCEAC, and TEAC values. More details are given in Table S1 (in the Supplementary Materials).

#### 2.1.2. Determination of IC_50_, VCEAC, and TEAC Values

In this work, the scavenging activity on DPPH^•^ involved 60 μL of the total analytical volume per sample including 10 μL of DPPH^•^ solution, 40 μL of ethanol, and 10 μL of a sample. The movement of the sample drop to mix with the dropped DPPH^•^ solution on the detection zone relied on the ethanol drop, which resembles a carrier in flow injection analysis systems. Waste generation was lower compared to the previous method that uses FIA and multisyringe flow injection analysis (MSFIA) [11,13]. In FIA, the DPPH^•^ solution is used as a reagent and carrier in one, and the injection volume of the sample is 50 μL. MSFIA employs 50 μL of the sample, 750 μL of DPPH^•^ solution, and 1500 μL of ethanol for carrying the sample, but still does not include the carrier needed to wash the system, about 1850 μL per analytical repetition.

**Figure 1:**
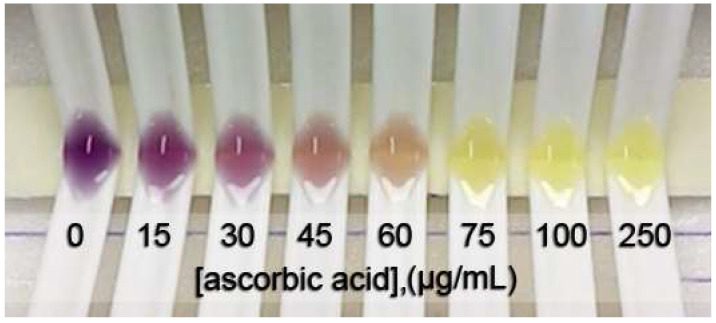
A photo of the color change of DPPH^•^ due to the various concentrations of L-ascorbic acid indicating DPPH^•^ scavenging activity.

**Figure 2:**
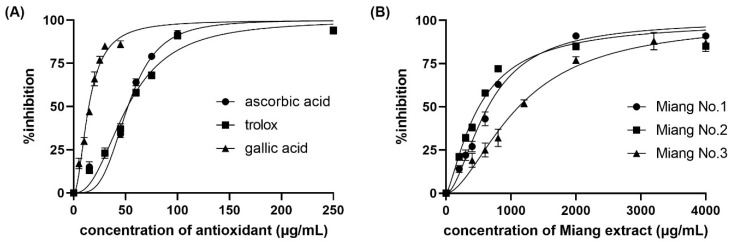
Percentage of DPPH^•^ inhibition against (**A**) concentration of positive control antioxidants, and (**B**) real samples.

Figure 1 shows the color change of the DPPH^•^ inhibition with different concentrations of L-ascorbic acid at steady state. The IC_50_ was obtained from a sigmoid curve plotted between the percentage of DPPH^•^ inhibition and the positive control or tested sample concentration. The sigmoid curve was fitted and indicated the IC_50_ using GraphPad Prism 9 software. The IC_50_ values of L-ascorbic acid, Trolox, and gallic acid are reported as the positive control and compared with the spectrophotometric method. The obtained sigmoid curves exhibiting the scavenging activity of positive controls are illustrated in Figure 2. A comparison between the proposed and reference method showed no statistically significant difference in the IC_50_ values (as shown in Table 1) of the positive control and samples. Furthermore, the relative standard deviation (RSD) in triplicate was less than 5%, indicating that the proposed method is precise.

Apart from the IC_50_, two other reported values are the VCEAC and TEAC. The percentage of DPPH^•^ inhibition was plotted against the concentrations of either L-ascorbic acid or Trolox to create calibration curves. A comparison between the proposed and reference methods revealed the VCEAC and TEAC of ascorbic acid, Trolox, and gallic acid, as shown in Table 1, in accordance with other previous reports [24,25,26]. The sample throughput was 6 samples/h/platform for IC_50_ measurement, and the high-throughput operation was 60 samples/h/platform for VCEAC and TEAC.

### 2.2. Total Phenolic Content Assay

The Folin–Ciocalteu (FC) assay was used to determine the total phenolic content (TPC) and is expressed as gallic acid equivalents (GAE). Dissociation of the phenolic proton of an antioxidant leads to a phenolate anion, which reacts with the FC reagent (containing phosphomolybdic/phosphotungstic acid) through a redox reaction under an alkaline environment to generate a blue molybdenum tungsten complex. The total phenolic content was determined by the proposed method using 100 μL of total analytical volume per sample, which included 40 μL of sodium carbonate solution, 50 μL of Folin–Ciocalteu reagent, and 10 μL of a sample. The previously reported methods operated using Folin–Ciocalteu reagent and sodium carbonate solution as the carrier. The Folin–Ciocalteu carrier flowed to merge the sample and continuously flowed to react with the sodium carbonate carrier. After that, it was incubated at the reaction coil before being pushed for detection. Leamsomrong et al. reported that the flow rate of each carrier was set to 1.9 mL/min, the injection volume of the sample was 300 μL, and the sampling throughput was 32 samples/h. That means the total analytical volume was 7.3 mL/sample, including 3.5 mL of Folin–Ciocalteu reagent, 3.5 mL of sodium carbonate solution, and 0.3 mL of sample in their work [27]. Another work published by Yoo et al. reported the use of the Folin–Ciocalteu carrier and sodium carbonate solution at the rate of 0.7 and 2.1 mL/min, respectively. It was found that, on average, the total analytical volume per sample was 3.5 mL/sample [28].

**Figure 3:**
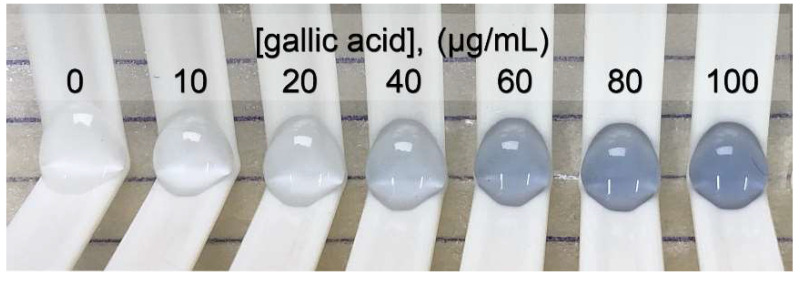
Photo of a blue product series providing a standard graph for determining the total phenolic content.

The concentration of gallic acid was plotted with the relative intensity of red (Δ intensity of red) for creating the standard linear range of 0 to 100 μg/mL. The linear equation was y = 1.5292x + 2.0992, r^2^ = 0.998, with a *p*-value < 0.05. The limit of detection and the limit of quantitation were 4.0 and 13.3 μg/mL, respectively. The signal, as the intensity of red, was extracted from a photo of the blue products, as shown in Figure 3, obtained from the oxidation–reduction reaction between phenolic compounds and Folin–Ciocalteu reagent. The high-throughput operation was performed with 40 samples/h/platform.

### 2.3. Application and Validation

**Figure 4:**
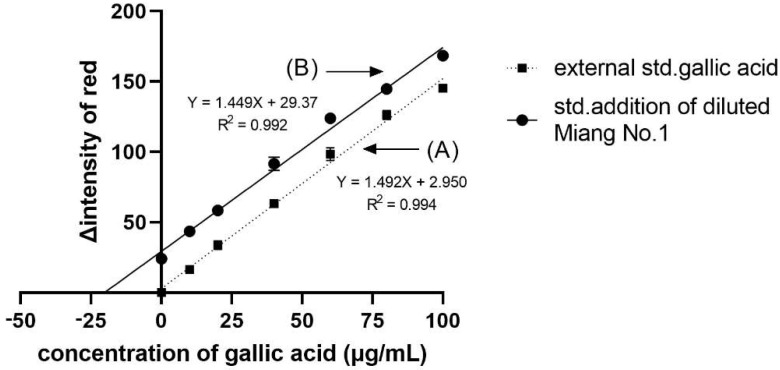
Linear regression plots of (**A**) the external standard calibration and (**B**) the standard addition calibration curves of the diluted Miang extract No.1. The error bars are standard deviations.

From the application of DPPH^•^ scavenging assay to Miang (fermented tea leaves) extracts using the proposed method, we obtained the IC_50_, VCEAC, and TEAC values compared to the reference method, as summarized in Table 1. Such antioxidant activity occurred from the antioxidant compounds in Miang that were reported in a previous work [29]. Figure 2B shows the obtained sigmoid curves exhibiting the scavenging activity of Miang extracts. These results are not significantly different at a confidence level of 95% comparing both methods, representing high precision with an RSD incidence of less than 5%. Moreover, the total phenolic content assay application to the Miang samples was based on the standard addition method to avoid matrix interference. Using different types or brands of digital cameras, optimization of parameters for image processing would be required before further routine operation. Another effect was video mode to the reaction time. In this study, the change due to reactions can be observed on a min scale that can use normal mode on a smartphone. Using the normal video mode for monitoring and the slow mode of some software for converting a video to a photo fast reaction (reaction change on seconds scale) was reported in the previous work [20]. However, using a slow mode available in some smartphones is of interest and would be plan in future study. Figure 4 shows that the linear regressions of the external standard calibration graph and the standard addition graph of the diluted Miang extract No.1 were parallel each other. That indicated there was no matrix effect in the Miang extract. The total phenolic content in the sample is the point where extrapolation of the graph touches the *x*-axis. The results obtained from the proposed method and that of the reference method are presented in Table 2. It was found that no significant differences were observed using the *t*-test at a confidence level of 95%.

From the investigation into the antioxidant activity in the Miang extracts, we found a reasonable relation between IC_50_, VCEAC, TEAC, and TPC. A comparison among the three samples exhibited the highest antioxidant activity in Miang No.2, which was observed from the lowest of IC_50_ and the highest of VCEAC and TEAC. Additionally, the antioxidant activity via DPPH^•^ scavenging is related to TPC because DPPH^•^ scavenging occurs by donating a hydrogen atom from the phenolic OH to the DPPH^•^ (the hydrogen atom transfer (HAT) mechanism) [30]. Another mechanism for free radical scavenging involves single electron transfer–proton transfer (SET-PT) by an electron transported from PhOH to the radical. The obtained results according to such theory showed the highest antioxidant activity for free radical scavenging and the highest TPC in Miang No.2. Conversely, decreases in antioxidant activity and TPC were found in Miang extract No.1 and No.3, respectively.

**Table 2:** Total phenolic content of Miang (fermented tea leaf) extracts.

Samples	TPC (GAE; mg/g Sample)	
	Proposed Method	Reference Method [31]
Miang No.1	24 ± 2	25.2 ± 0.5
Miang No.2	32 ± 2	32 ± 1
Miang No.3	14 ± 2	12 ± 1

## 3. Materials and Methods

### 3.1. Reagents and Materials

All chemicals used were analytical grade; L-ascorbic acid, sodium carbonate (Thermo Fisher Scientific, Waltham, MA, USA), and ethanol (QRëC, Auckland, New Zealand) were employed. In addition, gallic acid monohydrate, 2,2-diphenyl-1-picrylhydrazyl, Folin–Ciocalteu reagent, and Trolox were obtained from Merck, Germany.

### 3.2. Fabrication of the Downscaling Platform Based on a Moving Drop

**Figure 5:**
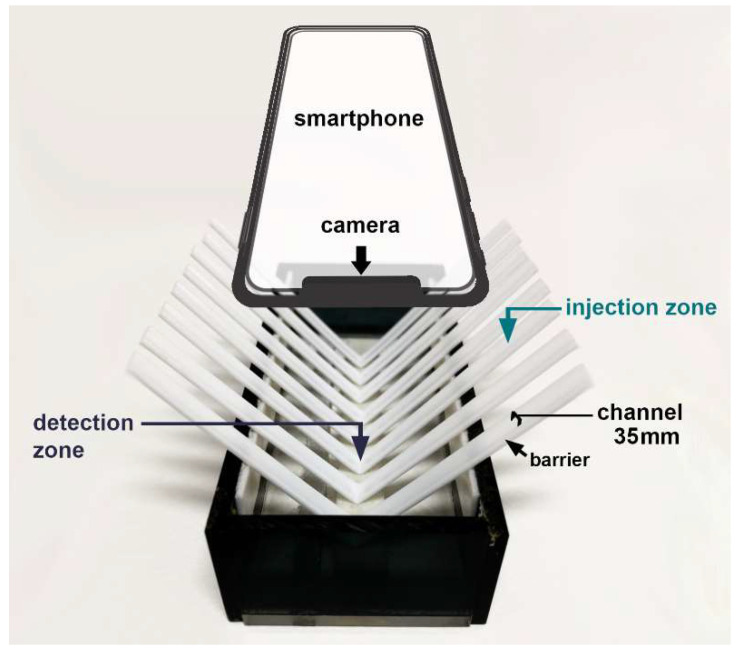
The minimized flow system is based on moving drops and a smartphone camera as the detector.

The platform, modified from [20], was a box with dimensions of 5.0 × 12.0 × 2.5 cm containing the arranged channels, as illustrated in Figure 5. The V-shaped channel was fabricated by folding a flat plastic coffee stirrer in half. The stirrer tubes were rinsed with deionized water and left to dry before use. Its cross-section showed a bottom surface parallel to the horizontal line and a semicircular curved top surface, creating the barrier sides. The lowest part of the V-shaped channel and both wings were designed as the detection zone and injection zone, respectively. The position of the injection zone was longitudinally above the detection zone of 5.0 cm. The longitudinal barrier of a channel can control the movement with the gravitational flow of the drop solution.

### 3.3. Scavenging Activity on 2,2-Diphenyl-1-Picrylhydrazyl (DPPH^•^) Radicals

The measurement of antioxidant activity via the scavenging effects on DPPH^•^ was modified from the previous report of Lai et al. [32]. Initially, 10 μL of 1 mM DPPH^•^ solution was dropped on the detection zone. Secondly, 10 μL of the positive control or tested samples was placed on the injection zone where the drop could adhere because the surface tension of the channel overcame the gravity acting on the drop. Then, the drop was eluted to move toward the detection zone by dropping 40 μL of ethanol above the positive control or tested samples drop. The continuous scavenging reaction was monitored using the general video mode (no filter) of the smartphone camera (ASUS Zenfone 6, Taipei, Taiwan). The recorded video resolution was 720 p (1280 × 720 pixels), and it was recorded with 20 frames/second. Then, the video was converted to photos using VLC media player and translated into color signals using Adobe Photoshop^®^ software to study the kinetics and scavenging effects on DPPH^•^. Adobe Photoshop^®^ software was selected for the evaluation, as it already existed for other office uses. Other free wares/apps should also serve the purposes. The ratio of magenta intensity to yellow intensity was used to calculate the percentage of DPPH^•^ inhibition following Equation (1). The spectrophotometric method was used as the reference method. Briefly, 1 mL of 0.2 mM DPPH^•^ in ethanol was reacted with 0.2 mL of extract. The DPPH^•^ scavenging activity was indicated by a decrease in DPPH^•^ absorbance at 515 nm.

### 3.4. Total Phenolic Content by the Folin–Ciocalteu Reaction

The developed downscaling TPC determination based on the Folin–Ciocalteu reaction was modified from that previously reported by Singleton [33]. Initially, 10 μL of the positive control or sample solutions was dropped in the detection zone. Then, 40 μL of 0.7 M sodium carbonate solution and 50 μL of 0.2 N Folin–Ciocalteu reagent were dropped on the injection zone and above the detection zone at about 5.0 cm, respectively. The reaction mixture was incubated for 15 min before a photo was taken and the color signals were translated by ImageJ software. The intensity of red was interpreted to the relative color intensity with blank (Δintensity of red) as color signals to create a calibration curve. The spectrophotometric method was used as the reference method [31]. Briefly, 0.05 mL of positive control or tested samples was mixed with 0.05 mL of 2.0 N Folin–Ciocalteu reagent and incubated for 6 min. After that, 0.4 mL of deionized water and 0.5 mL of 7% sodium carbonate solution were added. The reaction mixture was incubated for 30 min in the dark, and the absorbance was determined at 760 nm.

### 3.5. Application to the Miang (Fermented Tea Leaf) Extract

The fermented tea leaves were dried at 60 °C in a hot air oven for 24 h and ground into a coarse powder. Two grams of dry Miang powder was weighed and extracted with 50 mL deionized water at 60 °C for 1 h. The Miang extract was filtrated with Whatman filter No.1 before investigating. The filtrate was prepared to a concentration range of 0–4000 μg/mL for the DPPH^•^ scavenging assay. For the total phenolic content assay, the filtrated Miang extracts No.1 and No.2 were diluted with deionized water 50-fold, and No.3 was diluted 25-fold.

## 4. Conclusions

The downscaling platform based on a moving drop for antioxidant analysis was easily fabricated to be compatible with a smartphone detector. The proposed minimized system, which provides solution handling with flow similar to the flow analysis techniques, was found to be suitable for studying the kinetics of the 2,2-diphenyl-1-picrylhydrazyl radical scavenging activities to estimate the half-maximal inhibitory concentration (IC_50_), vitamin C equivalent antioxidant capacity (VCEAC), and Trolox equivalent antioxidant capacity (TEAC) at steady state. We succeeded in measuring the antioxidant activity in Miang extract. Furthermore, the total phenolic content assay was demonstrated. The developed method can provide numerical results with excellent accuracy and precision. It is cheaper, simpler, and smaller than the conventional method; represents a cost-effective green chemical analysis; and can be deployed in any environment and situation.

## Figures and Tables

**Table 1:** IC_50_, VECAC, and TEAC of the positive controls and real samples.

Antioxidants	IC_50_ (μg/mL)		VCEAC		TEAC	
	Proposed Method	Reference Method	Proposed Method	Reference Method	Proposed Method	Reference Method
Ascorbic acid	52 ± 1	53.0 ± 0.3	1.00 ± 0.03	1.00 ± 0.01	0.99 ± 0.10	0.98 ± 0.02
Trolox	52 ± 2	49.0 ± 0.3	1.04 ± 0.07	1.04 ± 0.03	1.00 ± 0.06	1.00 ± 0.03
Gallic acid	14 ± 1	15 ± 1	2.63 ± 0.10	2.60 ± 0.09	2.40 ± 0.1	2.50 ± 0.08
Miang No.1	618 ± 37	638 ± 5	0.36 ± 0.03 ^1^	0.35 ± 0.01 ^1^	0.36 ± 0.03 ^2^	0.33 ± 0.01 ^2^
Miang No.2	409 ± 18	425 ± 8	0.40 ± 0.04 ^1^	0.38 ± 0.01 ^1^	0.39 ± 0.04 ^2^	0.37 ± 0.01 ^2^
Miang No.3	1119 ± 56	1063 ± 44	0.18 ± 0.02 ^1^	0.18 ± 0.01 ^1^	0.18 ± 0.02 ^2^	0.17 ± 0.01 ^2^

^1^ mmole ascorbic acid/g sample, ^2^ mmole Trolox/g sample.

## Data Availability

The data presented in this study are available on request from the corresponding author.

## Supplementary Materials

The following are available online, Figure S1: Time-dependent of the inhibition percentage on 2,2-diphenyl-1-picrylhydrazyl radical by L-ascorbic acid. Figure S2: Time-dependent of the inhibition percentage on 2,2-diphenyl-1-picrylhydrazyl radical by Trolox. Figure S3: Time-dependent of the inhibition percentage on 2,2-diphenyl-1-picrylhydrazyl radical by gallic acid. Figure S4: Time-dependent of the inhibition percentage on 2,2-diphenyl-1-picrylhydrazyl radical by Miang extract sample No.1. Figure S5: Time-dependent of the inhibition percentage on 2,2-diphenyl-1-picrylhydrazyl radical by Miang extract sample No.2. Figure S6: Time-dependent of the inhibition percentage on 2,2-diphenyl-1-picrylhydrazyl radical by Miang extract sample No.3. Table S1: The parameters of the proposed method in comparison to the conventional spectrophotometric method and the previous method.

## Sample Availability

Samples of the compounds are available from the authors.
