# Supplementary material for: A Simple Minimized System Based on Moving Drops for Antioxidant Analysis Using a Smartphone
Source: Molecules. 2021 Sep 22;26(19):5744. doi: 10.3390/molecules26195744 (PMC8510342; doi:10.3390/molecules26195744)
Supplement: Supplementary file 1 [file molecules-26-05744-s001.zip › molecules-1369041-supplementary.pdf]

A Simple Minimized System Based on Moving Drops for Antioxidant Analysis Using a Smartphone

Sutasinee Apichai 1,2,3, Kajorngai Thajee 3, Thanawat Pattananandecha 1,2,3, Chalermpong Saenjum 1,2,3,* and Kate Grudpan 2,3,4,*

1 Department of Pharmaceutical Sciences, Faculty of Pharmacy, Chiang Mai University, Chiang Mai 50200, Thailand; sutasinee.apichai@gmail.com (S.A.); thanawat.pdecha@gmail.com (T.P.)

2 Cluster of Excellence on Biodiversity-Based Economics and Society (B.BES-CMU), Chiang Mai University, Chiang Mai 50200, Thailand

3 Center of Excellence for Innovation in Analytical Science and Technology (I-ANALY-S-T), Chiang Mai University, Chiang Mai 50200, Thailand; nuibct@gmail.com (K.T.)

4 Department of Chemistry, Faculty of Sciences, Chiang Mai University, Chiang Mai 50200, Thailand

* Correspondence: chalermpong.s@cmu.ac.th (C.S.); kgrudpan@gmail.com (K.G.); Tel.: +66-89-950-4227 (C.S.); +66-89-755-1994 (K.G.)

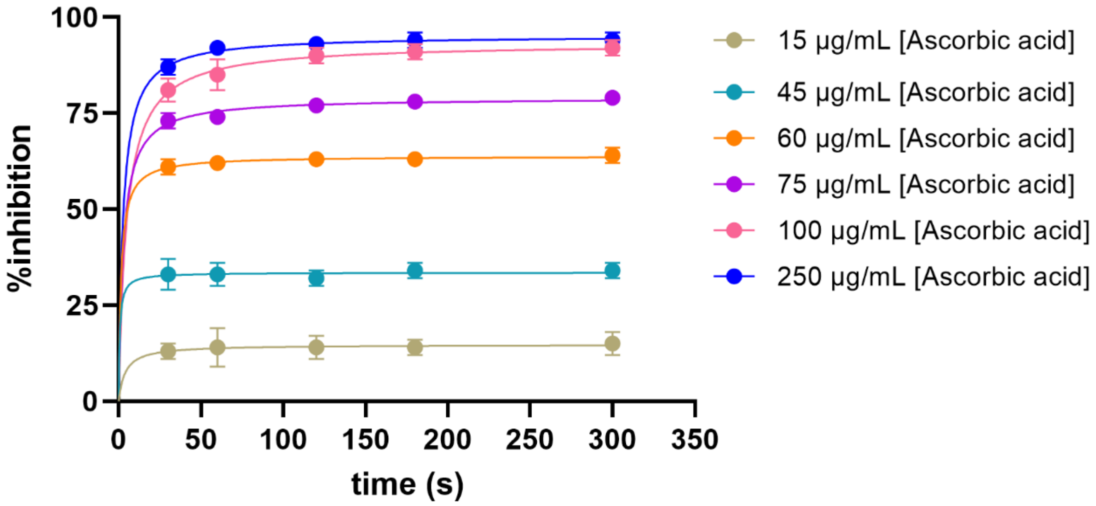

Figure S1. Time-dependent of the inhibition percentage on 2,2-diphenyl-1-picrylhydrazyl radical by L-ascorbic acid.

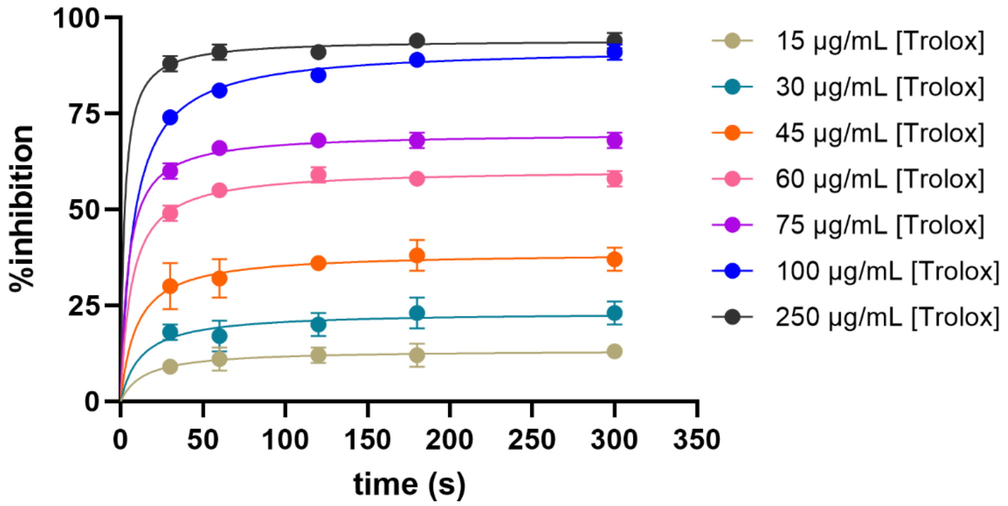

Figure S2. Time-dependent of the inhibition percentage on 2,2-diphenyl-1-picrylhydrazyl radical by Trolox.

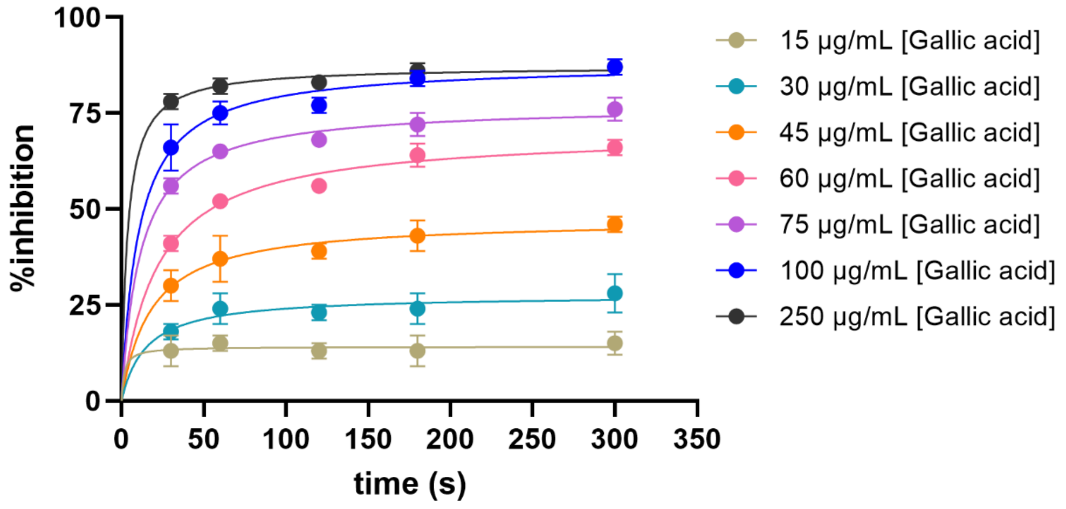

Figure S3. Time-dependent of the inhibition percentage on 2,2-diphenyl-1-picrylhydrazyl radical by gallic acid.

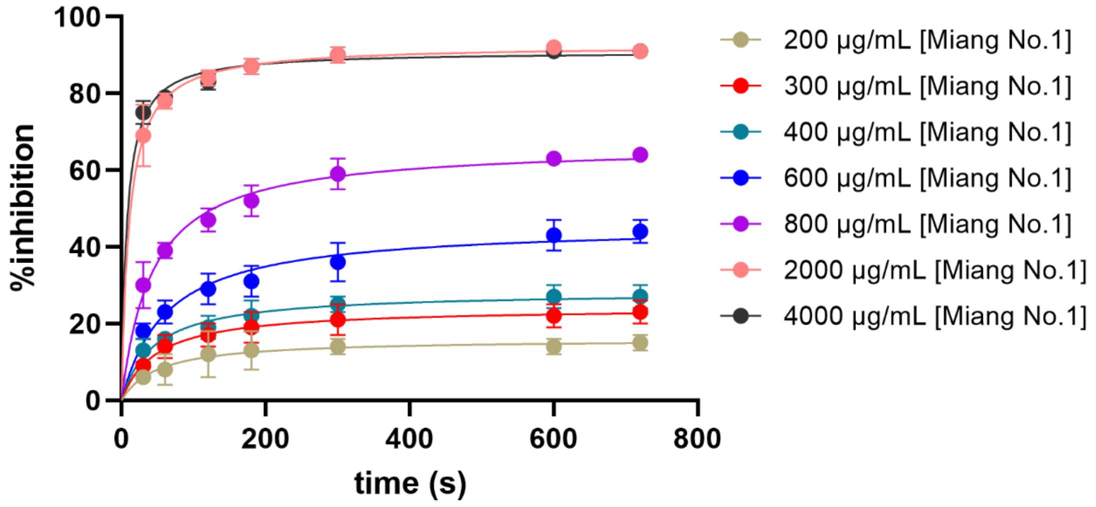

Figure S4. Time-dependent of the inhibition percentage on 2,2-diphenyl-1-picrylhydrazyl radical by Miang extract sample No.1.

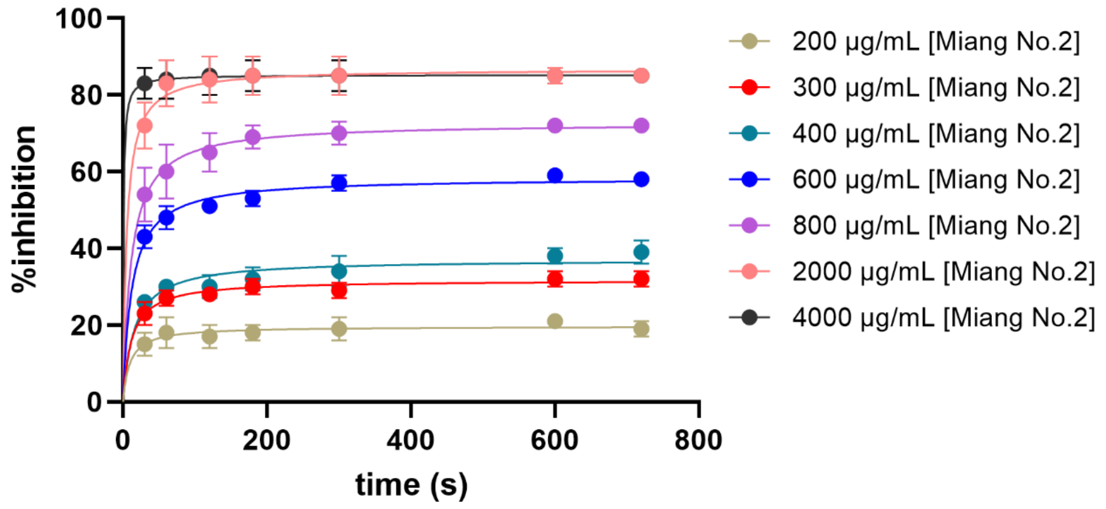

Figure S5. Time-dependent of the inhibition percentage on 2,2-diphenyl-1-picrylhydrazyl radical by Miang extract sample No.2.

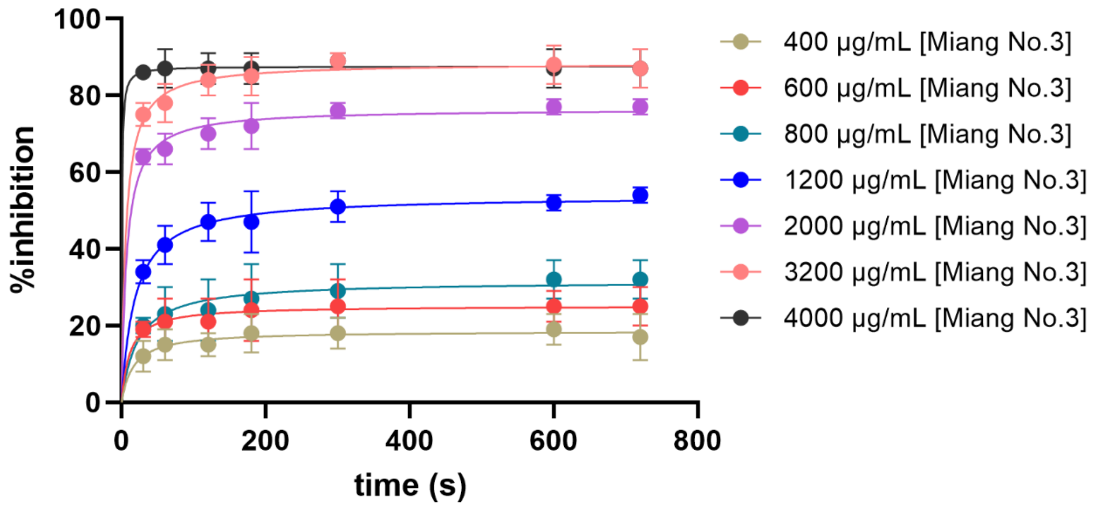

Figure S6. Time-dependent of the inhibition percentage on 2,2-diphenyl-1-picrylhydrazyl radical by Miang extract sample No.3.

Table S1. The parameters of the proposed method in comparison to the conventional spectrophotometric method and the previous method.

Title	Reference method [32]	The previous method [17]	The proposed method
Chemistry	Reduction reaction of 2,2-diphenyl-1-picrylhydrazyl (DPPH•, purple) to 2,2-diphenyl-1-picrylhydrazine ($DPPH_2$, yellow) with antioxidant	Based on antioxidant react with HOCl and the rest HOCl react with DMMA-IR780 probe (blue) to pink compound	Reduction reaction of 2,2-diphenyl-1-picrylhydrazyl (DPPH•, purple) to 2,2-diphenyl-1-picrylhydrazine ($DPPH_2$, yellow) with antioxidant
Reported values	The percentage of DPPH• inhibition (IC_{50}, VCEAC and TEAC)	The percentage of HOCl inhibition	The percentage of DPPH• inhibition (IC_{50}, VCEAC and TEAC)
Stability of free radical	**Condition**: room temperature<br><br>On the other hand, some researchers used higher temperatures for the DPPH assay, such as 37°C or even 50°C.	**Condition**: Temp. 25°C<br><br>The stability of a hypochlorite solution is affected by several parameters, including the concentration of hypochlorite, pH, temperature, the presence of certain contaminants that catalyze decomposition, and light exposure.	**Condition**: room temperature<br><br>On the other hand, some researchers used higher temperatures for the DPPH assay, such as 37°C or even 50°C.
Detector	Spectrophotometer	Smartphone	Smartphone
Platform	cuvette	96 well plate	Moving drop platform (Flat plastic coffee stirrer)
Manipulation	volume with micropipette	Drop and volume with micropipette	Drop with micropipette
Volume analysis	1.2 mL<br><br>Sample: 200 µL<br>Reagents: DPPH• in ethanol 1,000 µL	200 µL<br><br>Sample: 25 µL<br>Reagents: HOCl 25 µL and probe 150 µL	60 µL<br><br>Sample: 10 µL<br>Reagents: DPPH• 10 µL and ethanol 40 µL
Detection time	20 minutes for chemical and sample	3 minutes for chemical	3 minutes for chemical<br>10 minutes for sample
Detection mode	Absorbance	Intensity if color in RGB mode	Intensity if color in CMYK mode

Title	Reference method [32]	The previous method [17]	Developed method
% Inhibition	$scavenging\ effect\ (\%) = \left[1 - \frac{A_s}{A_c}\right] x\ 100$<br><br>As: Absorbance of sample at 517 nm<br>Ac: Absorbance of control at 517 nm	$HCSC\ (\%) = 100\ x\ \frac{C_s - C_{Clo}}{C_0 - C_{clo}}$<br><br>$C_s$: the red to blue ratio of the mixture of HOCl, reagent probe and sample<br>C_o: the red to blue ratio of the reagent probe solution<br>C_{Clo}: the red mixture of HOCl and reagent probe	$Inhibition\ (\%) = \left[\frac{I_o - I_t}{I_0}\right] x\ 100$<br><br>$I_0$: the magenta to yellow ratio of control<br>I_t: the magenta to yellow ratio of sample
Sample throughput	1 sample/platform	80 sample/platform	10 sample/platform
Light effect	Control with the opaque instrument	Control with LED array	Control with LED array lightbox
Cost of device	Platform: 31 USD/sample (can be reused)<br>Reagents: 1 USD/3 samples	Platform: 1 USD/80 samples (can be reused)<br>Reagents: non-available in the market	Platform: 1 USD/200 samples (can be reused)<br>Reagents: 1 USD/100 samples
Application	Laboratory	On-site	On-site
